# Catalytic Properties of NADP-Reducing Enzymes from *Streptococcus cristatus* ATCC 51100

**DOI:** 10.3390/biom16081212

**Published:** 2026-08-20

**Authors:** Isabell Schütt, Jonathan Teuffel, Ben H. Hlawatschke, Philip Einwohlt, Bernd Kreikemeyer, Rebecca C. Wade, Tomas Fiedler

**Affiliations:** 1 Institute of Medical Microbiology, Virology, and Hygiene, Rostock University Medical Centre, Schillingallee 70, 18057 Rostock, Germany; isabell.schuett@uni-rostock.de (I.S.); philip.einwohlt@uksh.de (P.E.); bernd.kreikemeyer@med.uni-rostock.de (B.K.); 2 Molecular and Cellular Modeling Group, Heidelberg Institute for Theoretical Studies, 69118 Heidelberg, Germany; 3 Center for Molecular Biology of Heidelberg University (ZMBH), German Cancer Research Center (DKFZ)-ZMBH Alliance, 69117 Heidelberg, Germany; 4 Graduate School of Mathematical and Computational Methods for the Sciences (HGS MathComp), Heidelberg University, 69117 Heidelberg, Germany; 5 Interdisciplinary Center for Scientific Computing (IWR), Heidelberg University, 69117 Heidelberg, Germany; 6 Faculty of Engineering Sciences, Heidelberg University, 69117 Heidelberg, Germany

**Keywords:** streptococci, NADPH metabolism, GapN, pentose phosphate pathway, redox metabolism, enzyme kinetics, metabolic flexibility, comparative enzymology, homology modeling

## Abstract

*Streptococcus cristatus* (*S. cristatus*) belongs to the viridans group of streptococci and is a commensal of the human upper respiratory tract. With the non-phosphorylating glyceraldehyde-3-phosphate dehydrogenase, GapN, and the oxidative part of the pentose phosphate pathway (oxPPP), *S. cristatus* can use two different metabolic pathways to provide reduced nicotinamide adenine dinucleotide phosphate (NADPH), an essential cofactor of anabolic reactions such as fatty acid and amino acid biosynthesis. Regarding their NADP-reducing capacity, streptococci can be categorized into three groups: those that have only GapN, those that use only the oxPPP, and those that use both pathways. Here, we report on the experimental and computational characterization of the catalytic properties of the three NADP-reducing enzymes: GapN, glucose-6-phosphate dehydrogenase (G6PDH), and 6-phosphogluconate dehydrogenase (6PGDH) of *S. cristatus*. Kinetic analyses showed moderate substrate and cofactor affinities, with GapN displaying the tightest substrate binding, followed by 6PGDH and G6PDH, in agreement with structural and computational predictions. All three enzymes preferentially utilized NADP^+^, with only G6PDH exhibiting limited NAD^+^ promiscuity. Growth-phase-dependent activity patterns suggest dynamic adjustment of NADPH-generating pathways, with reduced GapN contribution and sustained oxPPP activity in the stationary phase. Regulatory screening indicated limited allosteric control, though feedback inhibition by NADPH and the ATP sensitivity of G6PDH point to conserved redox regulatory mechanisms. Comparative analysis across streptococci supports the concept that the coexistence of GapN and the oxidative pentose phosphate pathway in *S. cristatus* may provide metabolic flexibility by offering alternative routes for NADPH generation.

## 1. Introduction

Reduced nicotinamide adenine dinucleotide phosphate (NADPH) is an essential cofactor for maintaining the redox balance in all eukaryotic and prokaryotic cells. It serves as an electron donor in anabolic pathways, including the biosynthesis of DNA, amino acids, and lipids, and in the regeneration of the oxidative stress defense peptides glutathione and thioredoxin [1,2,3,4,5,6,7].

**Figure 1:**
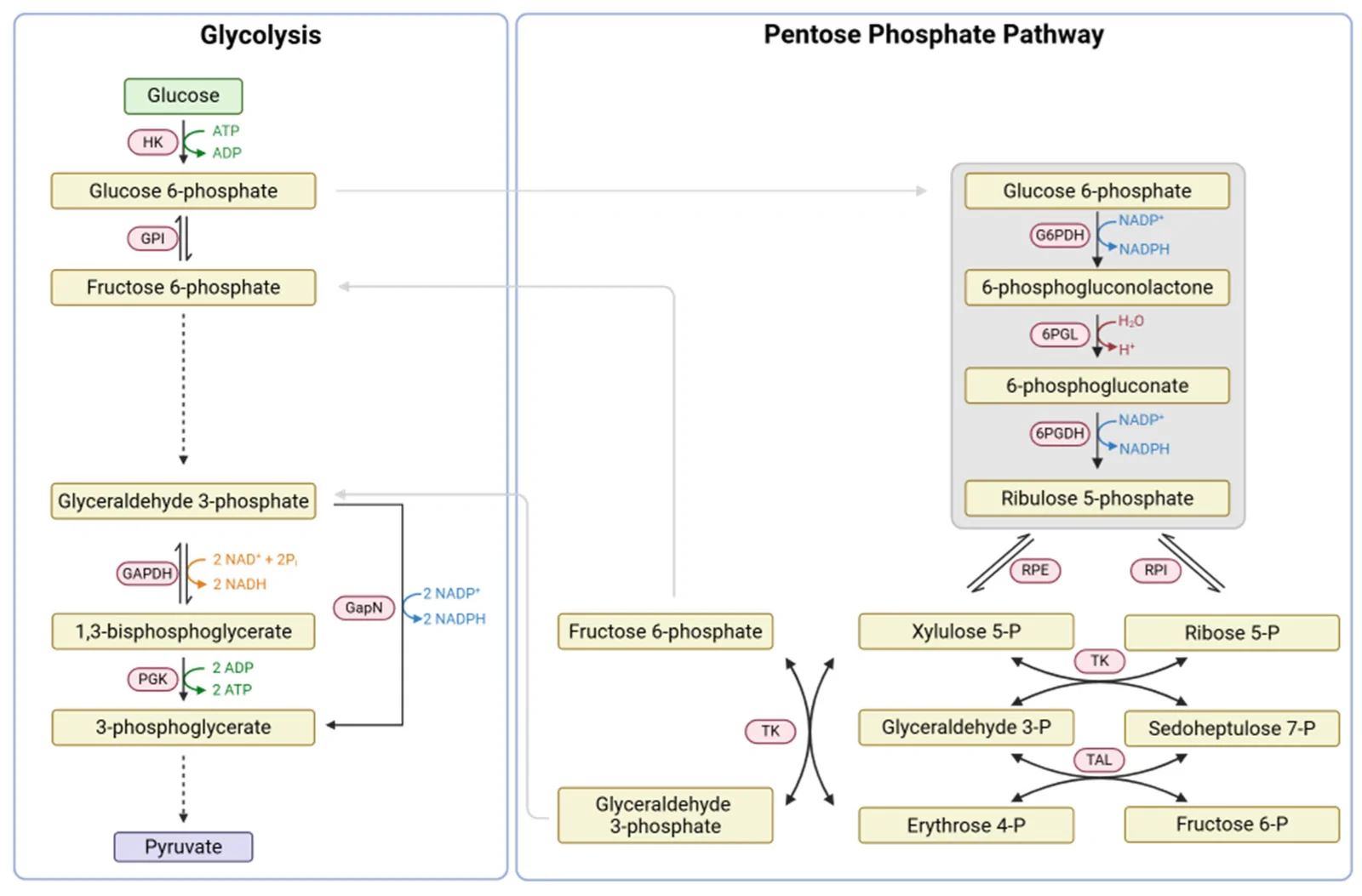
Schematic overview of glycolysis and the pentose phosphate pathway (PPP) in *S. cristatus*. The oxidative part of the PPP (oxPPP) is shaded in gray. HK, hexokinase; GPI, glucose-6-phosphate isomerase; GapDH, glyceraldehyde-3-phosphate dehydrogenase; PGK, phosphoglycerate kinase; GapN, non-phosphorylating glyceraldehyde-3-phosphate dehydrogenase; G6PDH, glucose-6-phosphate dehydrogenase; 6PGL, 6-phosphogluconolactonase; 6PGDH, 6-phosphogluconate dehydrogenase; RPE, ribulose-5-phosphate epimerase; RPI, ribose-5-phosphate isomerase; TK, transketolase; TAL, transaldolase. Created in BioRender. Einwohlt, P. (2026) https://BioRender.com/m0z8tr5.

In most bacteria, NADPH is obtained mainly via the pentose phosphate pathway (PPP), one of the first metabolic pathways to be discovered [8,9,10]. The first three PPP reactions form the oxidative part of the pathway (oxPPP) and are catalyzed by the enzymes glucose-6-phosphate dehydrogenase (G6PDH), 6-phosphogluconolactonase (6PGL), and 6-phosphogluconate dehydrogenase (6PGDH) [11,12,13] (Figure 1). G6PDH (EC 1.1.1.49) oxidizes D-glucose-6-phosphate to 6-phosphogluconolactone while reducing NADP^+^ to NADPH. 6PGL (EC 3.1.1.31) hydrolyzes 6-phosphogluconolactone to 6-phosphogluconate. 6PGDH (EC 1.1.1.44) catalyzes the oxidative decarboxylation of 6-phosphogluconate to ribulose-5-phosphate upon the reduction of NADP^+^ to NADPH. Since the PPP does not include reductive steps, it yields two molecules of NADPH per molecule of glucose-6-phosphate.

Another common way of providing NADPH is an alternative glycolytic route using the NADP^+^-dependent non-phosphorylating glyceraldehyde-3-phosphate dehydrogenase GapN, which was first identified in photosynthetic tissue [14] (Figure 1). GapN irreversibly converts glyceraldehyde-3-phosphate (G3P) to 3-phosphoglycerate (3-PG) while reducing NADP^+^ to NADPH [15,16]. By circumventing the glycolytic reactions of the conventional phosphate-dependent glyceraldehyde-3-phosphate dehydrogenase (GapDH) and phosphoglycerate kinase (PGK), it bypasses both NADH- and ATP-generating steps [11,17].

In streptococci, GapN was first described in *S. mutans* [16,18,19]. While most streptococcal species carry genes for both GapN and the oxPPP enzymes, some species like *S. pyogenes*, *S. mutans*, and *S. dysgalactiae* ssp. *equisimilis* possess GapN but lack the oxPPP. Other species, such as *S. mitis* and *S. suis*, have been shown to carry the oxPPP enzyme genes; however, GapN is not encoded in their genomes [20]. This suggests that at least one of the two metabolic pathways is required to ensure bacterial survival. GapN was already predicted to be an essential part of the *S. pyogenes* core genome based on gene essentiality analysis via transposon mutant libraries [21]. A bactericidal effect of a *gapN* knock-down using antisense peptide nucleic acids (asPNA) was previously observed in both *S. dysgalactiae* ssp. *equisimilis* and *S. pyogenes* [20]. This confirms the essentiality of GapN to these organisms, suggesting that GapN would be the major contributor to NADPH production, with no equivalent alternatives.

*S. cristatus* is equipped with both GapN and the oxPPP and, consequently, is not dependent on GapN, as previously demonstrated [20]. The species belongs to the Mitis group of streptococci and is generally associated with oral health [22,23,24,25]. It is predominantly found in supragingival plaque, although it has also been isolated from other sites within the oral cavity and the upper respiratory tract [26,27]. Although rare, *S. cristatus* has occasionally been reported to cause bacteremia and infective endocarditis [28,29,30,31].

This study provides a comprehensive biochemical and structural characterization of the three major NADP^+^-reducing enzymes in *S. cristatus*, GapN, G6PDH, and 6PGDH, and places them into a broader metabolic and phylogenetic context.

## 2. Methods

### 2.1. Strains and Culture Conditions

*S. cristatus* ATCC 51100 was grown in Brain Heart Infusion (BHI) medium at 37 °C under a 5% CO_2_-enriched atmosphere. *E. coli* DH5α and BL21 strains were grown in Lysogeny Broth (LB) medium at 37 °C under vigorous shaking in ambient air conditions. Recombinant *E. coli* strains carrying pASK-IBA2 or pASK-IBA7 derivatives (IBA GmbH, Philippsburg, Germany) were cultured in LB medium supplemented with ampicillin (100 mg/L, LB_Amp100_).

### 2.2. Construction of Expression Plasmids

Chromosomal DNA of *S. cristatus* ATCC 51100 was isolated using the DNeasy Blood and Tissue Kit (Qiagen, Hilden, Germany) following the manufacturer’s instructions for Gram-positive bacteria and used as a template for PCR amplification with the Phusion™ High-Fidelity DNA Polymerase and the primers listed in Table 1. All PCR products were purified using the QIAquick PCR purification kit (Qiagen). Amplicons of the *zwf* and *gndA* genes (encoding G6PDH and 6PGDH, respectively) were digested with BamHI and SalI and ligated into the equally treated pASK-IBA2 vector (IBA GmbH). *E. coli* DH5α cells were transformed with the recombinant vectors. Correct insertion of the respective PCR products was confirmed by Sanger sequencing (Eurofins Genomics, Ebersberg, Germany). For subsequent heterologous overexpression, *E. coli* BL21 cells were transformed with the recombinant plasmids.

### 2.3. Expression and Purification

Heterologous expression and purification of *S. cristatus* G6PDH (ScrG6PD) and 6PGDH (Scr6PGDH) were performed as described in Eisenberg et al. [20] using the recombinant *E. coli* strains constructed in this paper. For ScrGapN, *E. coli* pASK-IBA7_ScrGapN published in Schütt et al. [32] was used.

ScrGapN was dialyzed in 0.1 M citric acid (pH 6.0), while ScrG6PDH and Scr6PGDH were dialyzed in phosphate-buffered saline (PBS) (pH 7.4). Protein concentrations were determined using the Qubit protein BR assay kit following the manufacturer’s instructions (Thermo Fisher Scientific, Waltham, MA, USA).

### 2.4. SDS-PAGE and Western Blot Analysis

Sodium dodecyl sulfate-polyacrylamide gel electrophoresis (SDS-PAGE) was performed according to Laemmli [33]. Proteins on the SDS-PAGE gel were visualized using Coomassie Brilliant Blue. Electrophoretic transfer was carried out following the semi-dry method [34]. After blotting (45 min, 15 V), the membrane was blocked overnight in 1% skimmed milk diluted in PBS at 4 °C. The blot was washed using PBS. For chromogenic detection of Strep-tag proteins, the blot was incubated with Strep-Tactin alkaline phosphatase (AP) conjugate for 1 h while shaking. To remove excess conjugate, the membrane was washed with PBS. Subsequently, AP conjugate bound to Strep-tag proteins was detected using nitro-blue tetrazolium chloride/5-bromo-4-chloro-3′-indolyphosphate p-toluidine salt (NBT/BCIP) solution (Sigma-Aldrich, St. Louis, MI, USA).

### 2.5. Preparation of Protein Crude Extracts

To prepare protein crude extracts, BHI medium was inoculated 1:20 with *S. cristatus* overnight cultures. Bacterial growth was controlled by measuring the OD_600_. Samples were collected during the exponential (OD_600_ 0.5–0.55) and stationary (OD_600_ 1.2–1.3) phases and harvested by centrifugation (10 min, 4000× *g*). The pellet was resuspended in PBS (pH 7.4), and the cells were mechanically disrupted as described above. Cell debris was removed by centrifugation at 16,000× *g* and 4 °C for 10 min. Clear supernatant was collected, flash-frozen in liquid nitrogen, and stored at −20 °C. Protein concentrations were determined using the Qubit protein BR assay kit (Thermo Fisher Scientific, USA).

### 2.6. Enzymatic Assays

GapN Assay: The specific GapN activity was measured as described in Schütt et al. [32] based on the protocol of Iddar et al. [35].

G6PDH Assay: The specific G6PDH activity was measured as described in Haeussler et al. [36], with modification of the protocol. The assay mixture contained 10 mM NADP^+^, 100 mM Tris-HCl (pH 8.0), 10 mM MgCl_2,_ and 20 nM protein or 20 µL undiluted protein crude extract. The reaction was started by adding 6 mM G6P. The reduction of NADP^+^ to NADPH was detected by measuring absorbance at 37 °C and 340 nm in a spectrophotometer for up to 60 min. As controls, the assay was carried out without the addition of the protein sample and without the addition of G6P. Assays with a final concentration of 6 mM G3P and their respective controls were carried out using 200 mM Tricine pH 8.5.

6PGDH Assay: The specific 6PGDH activity was measured as described in Liu et al. [37], with modification of the protocol. The assay mixture contained 10 mM NADP^+^, 100 mM Tris-HCl (pH 8.0), 10 mM MgCl_2,_ and 100 nM protein or 20 µL undiluted protein crude extract. The reaction was started by adding 6 mM 6PG. The reduction of NADP^+^ to NADPH was detected by measuring absorbance at 37 °C and 340 nm in a spectrophotometer for up to 60 min. As controls, the assay was carried out without the addition of the protein sample and without the addition of 6PG. Assays with a final concentration of 6 mM G3P and their respective controls were carried out using 200 mM Tricine pH 8.5.

### 2.7. Quantitative Real-Time PCR

For RNA isolation, samples were collected from *S. cristatus* cultures during the exponential (OD_600_ 0.5–0.55) and stationary (OD_600_ 1.2–1.3) growth phases and centrifuged at 4 °C and 4000× *g* for 10 min. RNA was isolated using the FastRNA Pro Blue Kit (MP Biomedicals, Santa Ana, CA, USA), and remaining DNA was digested using a Turbo DNA-free Kit (Thermo Fisher Scientific, USA). Synthesis of cDNA was performed with the First Strand cDNA synthesis kit (Invitrogen, Carlsbad, CA, USA). For qPCR, primers listed in Table 2 were used with the Maxima SYBR Green/ROX qPCR Master Mix (Thermo Scientific, USA) on a LightCycler 480 II (Roche, Basel, Switzerland). A no-reverse transcription control was included for each RNA sample to verify the absence of genomic DNA contamination. The 16S-rRNA gene transcript was used for normalization. Relative gene expression was determined using the ΔΔCT method.

### 2.8. Statistical Analysis

The number of biological replicates (n) and the tests used to determine statistical significance for each data set are indicated in the respective figure captions. Statistical analyses were performed using GraphPad Prism 11.0.0 software.

### 2.9. Homology Modeling

Multimeric protein structures of the three protein sequences investigated in this study were generated through homology modeling using the SWISS-MODEL webserver [38]. Using the ‘Template Search’ function, a structure of the thioacyl intermediate of GapN from *S. mutans* at a resolution of 2.19 Å (PDB-ID: 2QE0, SeqID: 75.9%) was selected as the template for *S. cristatus* GapN (ScrGapN) [39]. The model of *S. cristatus* G6PDH (ScrG6PDH) was based on a crystallographic structure of a complex of an active Q365C mutant of *L. mesenteroides* G6PDH in a complex with NADP at a resolution of 2.16 Å (PDB-ID:1H9A, SeqID: 45.21%) [40]. Lastly, the model of *S. cristatus* 6PGDH (Scr6PGDH) was based on the structure of a 6PGDH from *L. lactis* at 2.4 Å (PDB-ID: 2IYO, SeqID: 74.1%) [41]. The quality of the generated models was assessed through the ‘Structure Assessment’ tools provided by the SWISS-MODEL webserver. Quality assessments of all models are given in Supplementary Table S1.

For ScrGapN, the NADP^+^ cofactors and native G3P substrate were inserted by alignment with and subsequent transfer from a holo-structure of *S. mutans* GapN (PDB-ID 1QI1) in PyMol [42,43]. To yield a holo-model of ScrG6PDH, the NADP^+^ cofactor was transferred from the template structure (PDB-ID: 1H9A) by superposition, and the G6P substrate was transferred from another structure of G6PDH from *L. mesenteroides* (PDB-ID: 1E77) [44]. For Scr6PGDH, SWISS-MODEL was able to include the 6PG substrate from the template structure (PDB-ID: 2IYO), and the NADP^+^ cofactor was inserted from alignment with another 6PGDH structure from *L. lactis* (PDB-ID: 2IZ0) [41]. Additionally, crystallographic cacodylate ions (CAC) from the 1IYO template that were also modeled by SWISS-MODEL were removed manually.

### 2.10. Molecular Docking Simulations

System and ligand preparation and molecular docking simulations were performed in the Maestro environment as implemented in the Schrödinger 2024r3 software package [45]. The docking workflow followed our previous work on *S. pyogenes* GapN (SpyGapN) described in Schütt et al. [32]. Briefly, the protein structures were imported into Maestro and prepared for docking with the ‘PrepWizard’ program, assigning protonation states at pH 7.0 ± 4.0 and correct bond orders and then performing a restrained minimization within the OPLS2005 force field. The three native substrates G3P, G6P, and 6PG were imported and set up for docking during the protein preparation protocol. Ligands that were not contained within the imported structures were drawn using the ‘2D-sketcher’ program and prepared separately using ‘LigPrep’ to assign protonation states with EpiK at pH 7.0 ± 3.0 and assign OPLS2005 parameters. Molecular docking was performed with GLIDE using the ‘Standard Precision’ docking procedure set to generate three poses per ligand and otherwise use default parameters [46]. Receptor grids were centered on the native substrate using default parameters.

## 3. Results

### 3.1. Structural Biology of GapN, G6PDH and 6PGDH

**Figure 2:**
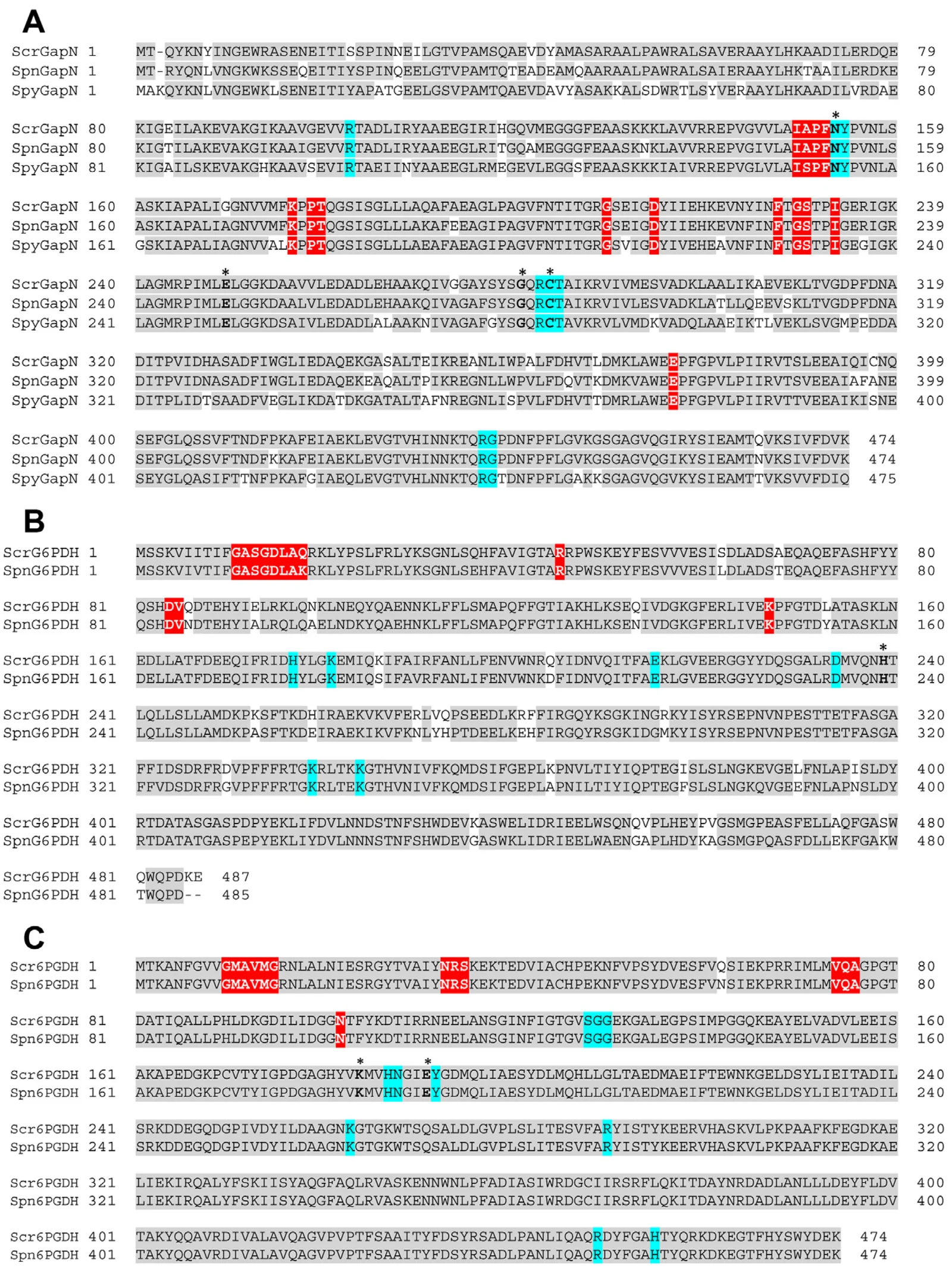
Amino acid sequence alignment of (**A**) GapN, (**B**) G6PDH, and (**C**) 6PGDH from *S. cristatus* (Scr) with the respective enzymes of *S. pneumoniae* (Spn) and *S. pyogenes* (Spy). Identical (functionally) amino acids are marked in gray. Key amino acids involved in substrate binding are marked in cyan. NADP-binding amino acids are marked in red. Catalytic residues are indicated by an asterisk.

GapN, G6PDH, and 6PGDH belong to the class of oxidoreductases and catalyze essential steps in NADPH generation. Amino acid sequence alignments of *S. cristatus* enzymes with homologous proteins from *S. pneumoniae* and *S. pyogenes* revealed conservation of NADP^+^-binding motifs, substrate-binding residues, and catalytic residues (Figure 2). The NADP^+^-binding sites include typical glycine-rich Rossmann fold motifs, which are usually conserved in NAD(P)-dependent oxidoreductases as they play a central role in cofactor binding [47,48].

The three-dimensional structures of all three enzymes were generated by homology modeling using related templates. Despite the moderate sequence identity between ScrG6PDH and its template from *L. mesenteroides* of only ~45%, all models showed high quality, as indicated by favorable QMeanDisCo scores and hardly any Ramachandran outliers (Supplementary Table S1). In all cases, the model quality was sufficient to support oligomeric states consistent with templates used for docking simulations (ScrGapN: tetramer, ScrG6PDH and Scr6PGDH: homodimer). All three proteins adopt different, mostly alpha-helical folds, with ScrGapN and ScrG6PDH featuring more beta-sheet-rich domains in proximity to the homodimerization interfaces.

**Figure 3:**
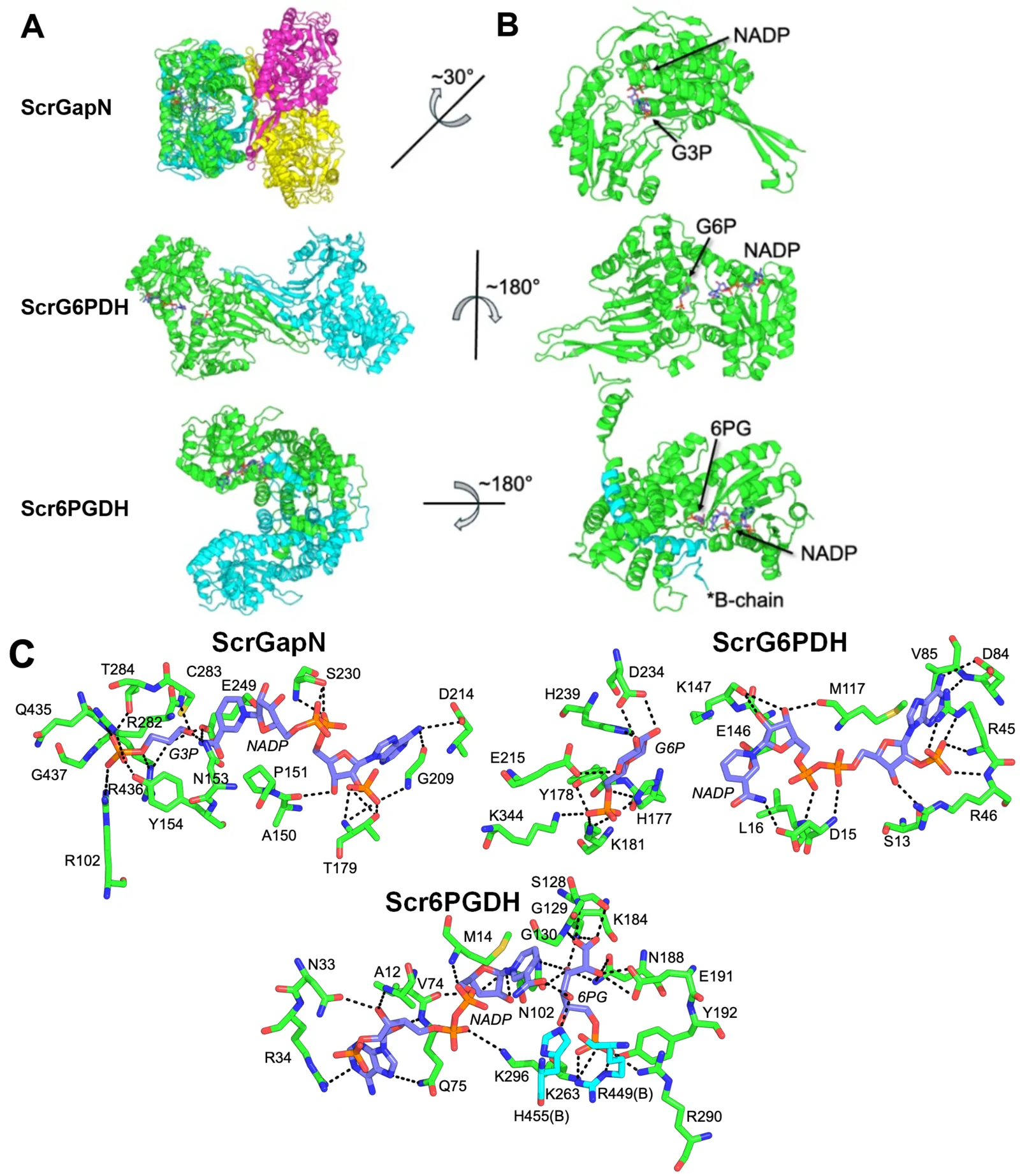
Homology models of the structures of *S. cristatus* GapN, G6PDH, and 6PGDH. In (**A**), the full homo-multimeric models used for the docking procedure are displayed. (**B**) displays one subunit per enzyme to highlight the location of the active sites and substrates (with the inclusion of the C-terminal part of the B-chain of 6PGDH, which forms part of the active site, indicated by an asterisk [*]). The subunits are rotated to highlight the orientations of the active sites as indicated by the approximate rotation axes. In (**C**), interacting residues and the bound substrates are displayed in ‘sticks’ representation and labeled accordingly. The proteins are color-coded by chain (A-chain: green, B-chain: cyan, C-chain: magenta, D-chain: yellow) and displayed in cartoon representation (**A**,**B**) or stick representation (**C**). The NADP and G3P, G6P and 6PG ligands are displayed in blue stick representation. In (**B**,**C**), only the A-chains of the homo-multimers are displayed, with the exception of 6PGDH, where a part of the active site is formed by the neighboring subunit, which is also shown and referred to as ‘B-chain’. Polar interactions are shown by black dashed lines.

As can be seen in Figure 3, the proteins’ active binding sites differ in solvent accessibility: G3P is fully immersed within a narrow, central tunnel in the active site of ScrGapN, whereas the active sites of ScrG6PDH and Scr6PGDH are located on the protein surfaces and rather exposed, indicating potentially lower specificity and affinity for the substrate compared to ScrGapN. In Scr6PGDH, a C-terminal helix intrudes into the neighboring subunit and forms half of the substrate-coordinating active site surface, consistent with the template structure. To correctly distinguish between interaction with ligands in the active site formed by either subunit, we henceforth refer to residues located in the C-terminal helix as ‘B-chain’ (cyan in Figure 3) and all others as ‘A-chain’ (green in Figure 3).

**Figure 4:**
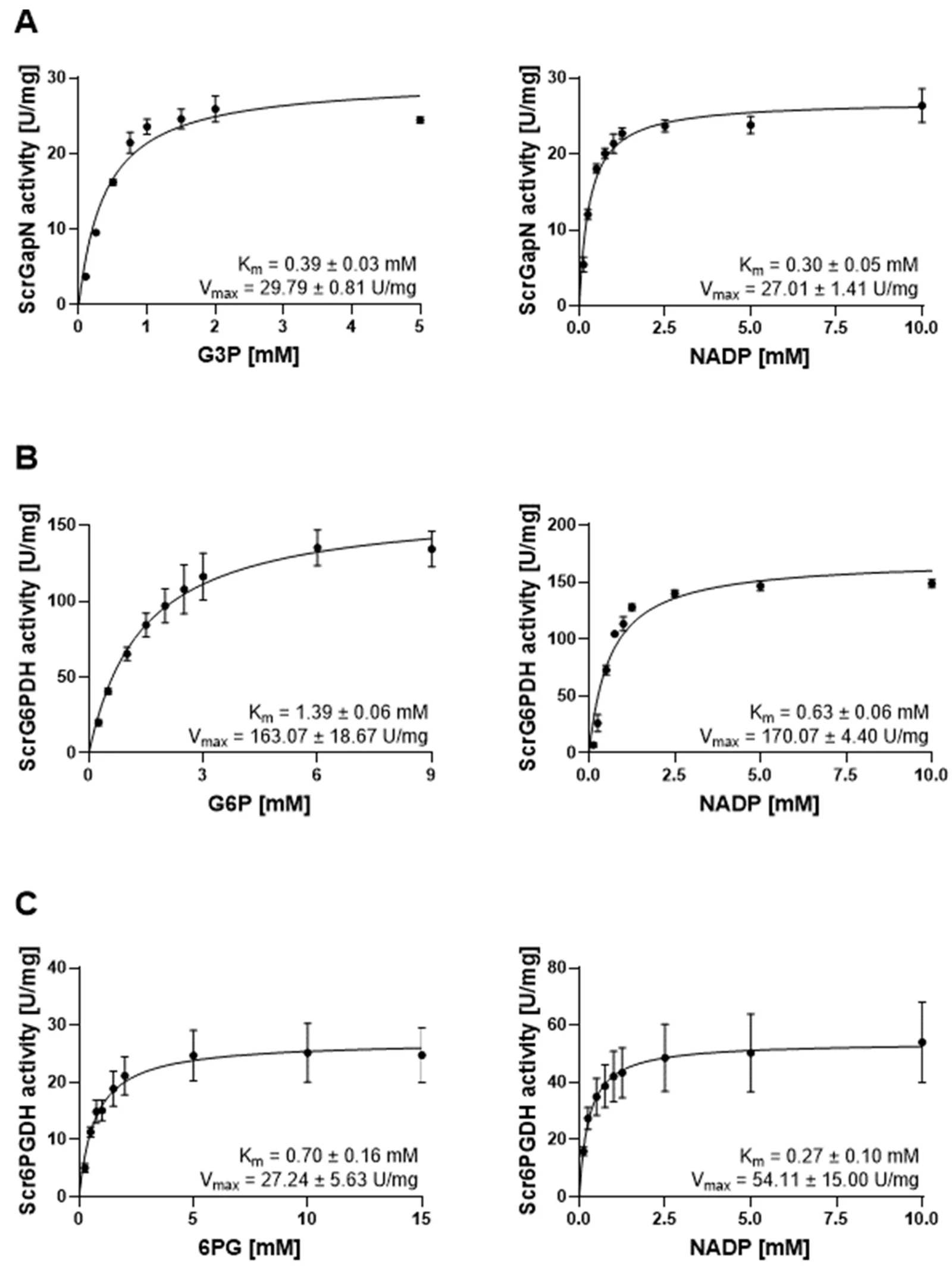
Michaelis–Menten plots. Kinetic parameters Km and Vmax were determined for different substrate (left column) and cofactor (right column) concentrations for purified (**A**) GapN, (**B**) G6PDH, and (**C**) 6PGDH from *S. cristatus*. For figures in the left column, the NADP concentration was fixed at 10 mM for each enzyme. For figures in the right column, the substrate concentrations were fixed at 2 mM for GapN and 6 mM for G6PDH and 6PGDH, respectively. Activities shown in U/mg protein. Km and Vmax values were projected values derived from regression analyses. Mean values and standard deviations, n = 3.

All active site residues described for SpyGapN [20] are conserved in ScrGapN. The G3P substrate’s phosphate moiety is tightly coordinated by three arginine residues (R102, R282 and R436) at the entrance of the binding site and forms further hydrogen bonds to Y154, T284, Q435 and G437. The G3P aldehyde group is positioned for oxidation in an oxyanion hole formed by N153 and the backbone amide of C283 and is in direct proximity to the nicotinamide moiety of the NADP^+^ cofactor (Figure 3). In ScrG6PDH, the distance between G6P and NADP^+^ binding sites is remarkably large, with the distance between the nicotinamide carbonyl-C and the site of oxidation (C1 in G6P) measuring 8.9 Å, indicating that conformational changes may be required for catalysis. G6P’s phosphate group is coordinated by Y178, H177, K181 and K344, whereas the carbohydrate hydroxyl moieties form hydrogen bonds to E215, D235 and H239. In contrast, the model of Scr6PGDH is the only one that features a direct hydrogen bond between the nicotinamide oxygen and a hydroxyl group of the substrate: the C4-hydroxyl group of 6PG. 6PG also makes a hydrogen bond via a hydroxyl group to H455 in the C-terminal helix of the B-chain. Additionally, the phosphate group is tightly coordinated by Y192, K263, R290 (A-chain) and R449 (B-chain). The C2- and C3 hydroxyl groups as well as the C1-carboxylic acid that form the opposite end of the substrate molecule with regard to the phosphate are involved in an intricate hydrogen-bonding network formed exclusively by the A-chain (N102, G129, G130, K184, N188, E191). The overall density of interactions with the substrate indicates that ScrGapN binds its native substrate most tightly, followed by Scr6PGDH and then ScrG6PDH, which is consistent with the measured Km values (Figure 4). Both ScrGapN and ScrG6PDH coordinate the O2’-phosphate of NADP^+^ via hydrogen-bonding networks, whereas this interaction is absent in the model of Scr6PGDH. In Scr6PGDH, nearby arginine R34 may adopt a different orientation in which it could coordinate this phosphate moiety. Overall, all three proteins have binding sites that appear to be rather specific for NADP^+^.

### 3.2. Kinetic Constants of GapN, G6PDH and 6PGDH

To assess whether the structural features are reflected in enzyme function, the kinetic properties of the three enzymes were determined experimentally. Michaelis–Menten kinetic constants for the respective substrates and cofactors were analyzed using the heterologously produced and purified enzymes (Supplementary Figure S1). The Michaelis–Menten plots and corresponding Km values are shown in Figure 4, while the calculated turnover numbers (Kcat) and catalytic efficiencies (Kcat/Km) are summarized in Table 3. Among the three enzymes, ScrG6PDH shows the lowest affinity to both its natural substrate, G6P, and NADP^+^ (Figure 4B), as indicated by the Km values of 1.39 ± 0.06 mM and 0.63 ± 0.06 mM, respectively. Despite this lower binding affinity, ScrG6PDH exhibited the highest turnover rates, with a Kcat of 80,630 ± 9231 s^−1^ for G6P and 84,091 ± 2176 s^−1^ for NADP^+^ resulting in catalytic efficiencies of (5.80 ± 0.71) × 10^7^ M^−1^ s^−1^ (G6P) and (1.33 ± 0.13) × 10^8^ M^−1^ s^−1^ (NADP^+^). As indicated by the in silico analysis, ScrGapN has the highest affinity for its natural substrate (Km = 0.39 ± 0.03 mM; Figure 4A), followed by Scr6PGDH (Km = 0.70 ± 0.16 mM; Figure 4C). Regarding these substrates, ScrGapN and Scr6PGDH displayed Kcat values of 6650 ± 181 s^−1^ and 12,723 ± 2630 s^−1^, yielding catalytic efficiencies of (1.71 ± 0.14) × 10^7^ M^−1^ s^−1^ and (1.82 ± 0.56) × 10^7^ M^−1^ s^−1^, respectively. The Km values of ScrGapN and Scr6PGDH for NADP^+^ were similar, with 0.30 ± 0.05 mM and 0.27 ± 0.10 mM, respectively, corresponding to a catalytic efficiency of (2.01 ± 0.35) × 10^7^ M^−1^ s^−1^ for ScrGapN and (9.36 ± 4.33) × 10^7^ M^−1^ s^−1^ for Scr6PGDH. Comparison of Km values with docking scores from re-docking of the native substrates (G3P, G6P, 6PG) showed a consistent trend, where ScrGapN:G3P yielded the most favorable average docking score of −8.00 ± 0.03 kcal/mol, followed by Scr6PGDH:6PG (−7.75 ± 0.53 kcal/mol), and ScrG6PDH:G6P (−6.21 ± 0.03 kcal/mol) (Table 4). This trend is consistent with the structural models, where tighter active sites and a higher density of substrate interactions correlate with more favorable binding affinities and lower Km values. The native poses of all substrates were obtained upon re-docking them into the homology models.

### 3.3. Impact of pH on GapN, G6PDH and 6PGDH Activity

**Figure 5:**
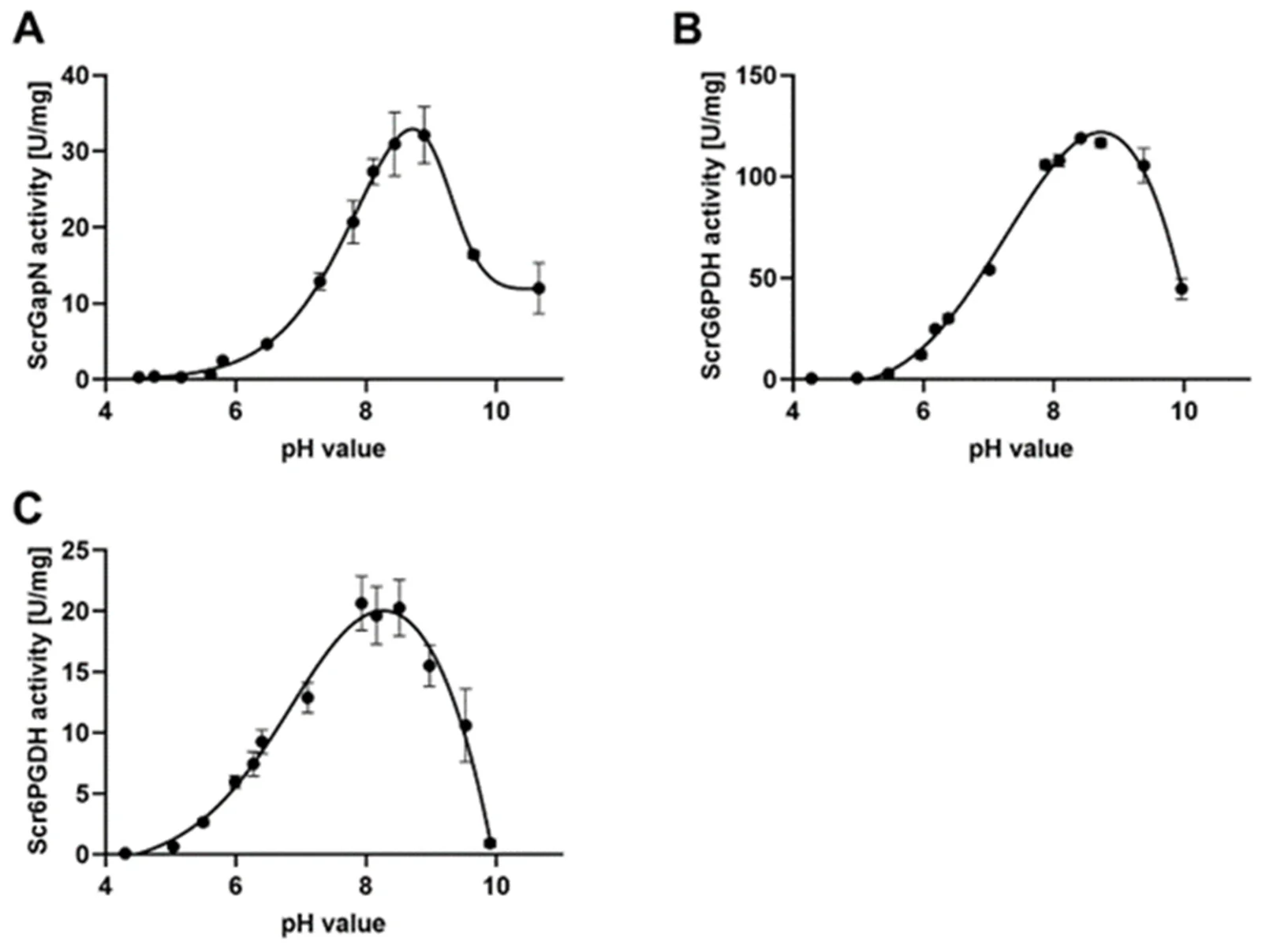
Impact of different pH values on activity of purified GapN (**A**), G6PDH (**B**), and 6PGDH (**C**) from *S. cristatus*. Activities shown in U/mg protein. Bell-curve fit. The reported pH optima correspond to the maxima of the fitted curves. Mean values and standard deviations, n ≥ 3.

Following kinetic characterization, the influence of pH on enzyme activity was examined. The pH dependence of the ScrGapN, ScrG6PDH, and Scr6PGDH activity was assessed across a pH range from 4 to 10.5 (Figure 5). None of the three enzymes showed detectable activity below pH 5. ScrGapN exhibited peak activity at pH 8 (Figure 5A), while ScrG6PDH and Scr6PGDH showed broader pH optima between pH 7.8 and 8.5 (Figure 5B,C). ScrGapN and ScrG6PDH retained activity up to pH 10.5 or pH 10.0, respectively, whereas no activity was observed for Scr6PGDH above pH 10. Overall, all three enzymes demonstrated a neutral-to-slightly alkaline pH optimum.

### 3.4. Impact of Potential Regulators of GapN, G6PDH and 6PGDH Through Competitive or Allosteric Binding

**Figure 6:**
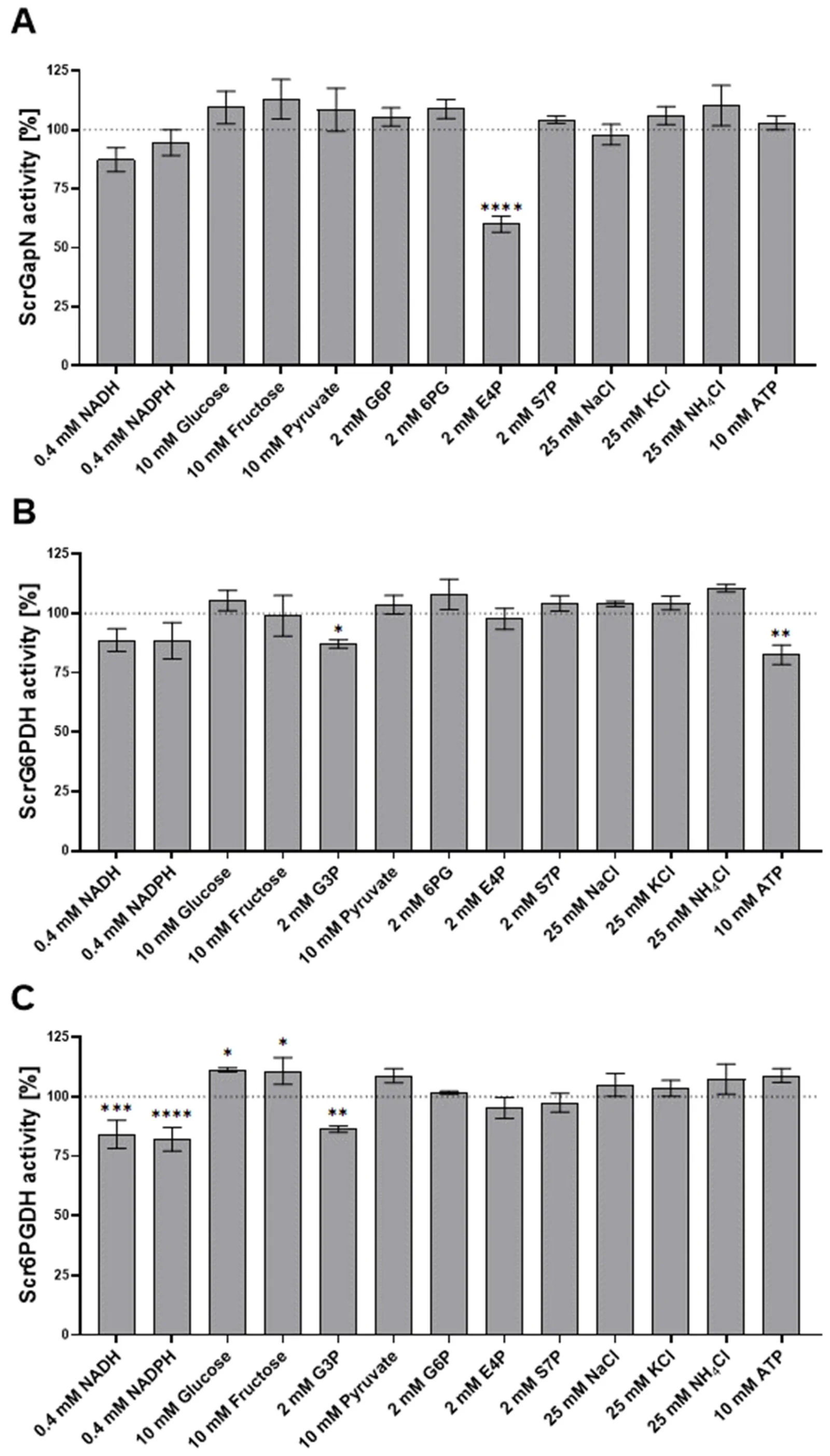
Influence of different salts and pathway intermediates tested at the given concentrations on purified (**A**) GapN, (**B**) G6PDH, and (**C**) 6PGDH from *S. cristatus*. Activities shown in % relative to an untreated control. Dotted line indicates 100% enzyme activity. Mean values and standard deviations, n = 3, one-way ANOVA, * *p* < 0.05, ** *p* < 0.01, *** *p* < 0.001, **** *p* < 0.0001.

In addition to pH, the effects of potential metabolic regulators on enzyme activity were investigated. Compounds previously reported to stimulate or inhibit NADPH-producing enzymes in other streptococci (*S. mutans*, *S. pyogenes*), or to exert feedback or feedforward regulation on ScrGapN, ScrG6PDH, and Scr6PGDH, were evaluated [16,20]. Tested compounds included reduced cofactors NADH and NADPH, key pathway intermediates of glycolysis (glucose, fructose, G3P and pyruvate), oxPPP (G6P and 6PG) as well as non-oxPPP (erythrose-4-phosphate, E4P, and sedoheptulose-7-phosphate, S7P), salts (NaCl, KCl, NH_4_Cl) and ATP [8,11] (Figure 6). In addition, the inhibitory potential of these compounds was assessed in silico by docking them to the structural models of ScrGapN, ScrG6PDH and Scr6PGDH (Table 3). Docking was limited to small-molecule competitive inhibitor candidates (G3P, G6P, 6PG, E4P, S7P, and pyruvate) due to the difficulties of assessing allosteric inhibitors when both the allosteric site and the allosteric mechanism are unknown. The docked compounds were classified according to the relative docking scores compared to the native substrate, the quality of the docking pose (considering potential clashes and the number of beneficial interactions), and whether the compound docked to the respective active site at all.

NADH and NADPH slightly inhibited all three enzymes, with a significant effect only for Scr6PGDH. Pyruvate, S7P, and salts showed no measurable effect, consistent with docking predictions indicating poor binding affinity or off-site docking (Table 3). For ScrGapN, S7P could not be accommodated in the active site and docked to a vestibular region, supporting the lack of inhibition. Similarly, ATP had no effect on ScrGapN, whereas it significantly inhibited ScrG6PDH. E4P reduced ScrGapN activity by around 40%, which was less than reported for SpyGapN [20]. Despite a fully conserved active site between SpyGapN and ScrGapN, E4P showed reduced inhibitory potential toward ScrGapN, consistent with docking results showing a >1 kcal/mol less favorable score compared to G3P, whereas docking to SpyGapN predicted a ~0.5 kcal/mol more favorable score for E4P relative to G3P (Table 3) [20]. Electrostatic repulsion may contribute to this effect, as ScrGapN carries a slightly more negative tetrameric net charge (−60 e) compared to SpyGapN (−52 e) (charges obtained from ‘PrepWizard’ upon assignment of protonation states). As E4P is also negatively charged under physiological conditions, a more negatively charged protein structure may display weakened attraction to the ligand due to increased electrostatic repulsion.

**Figure 7:**
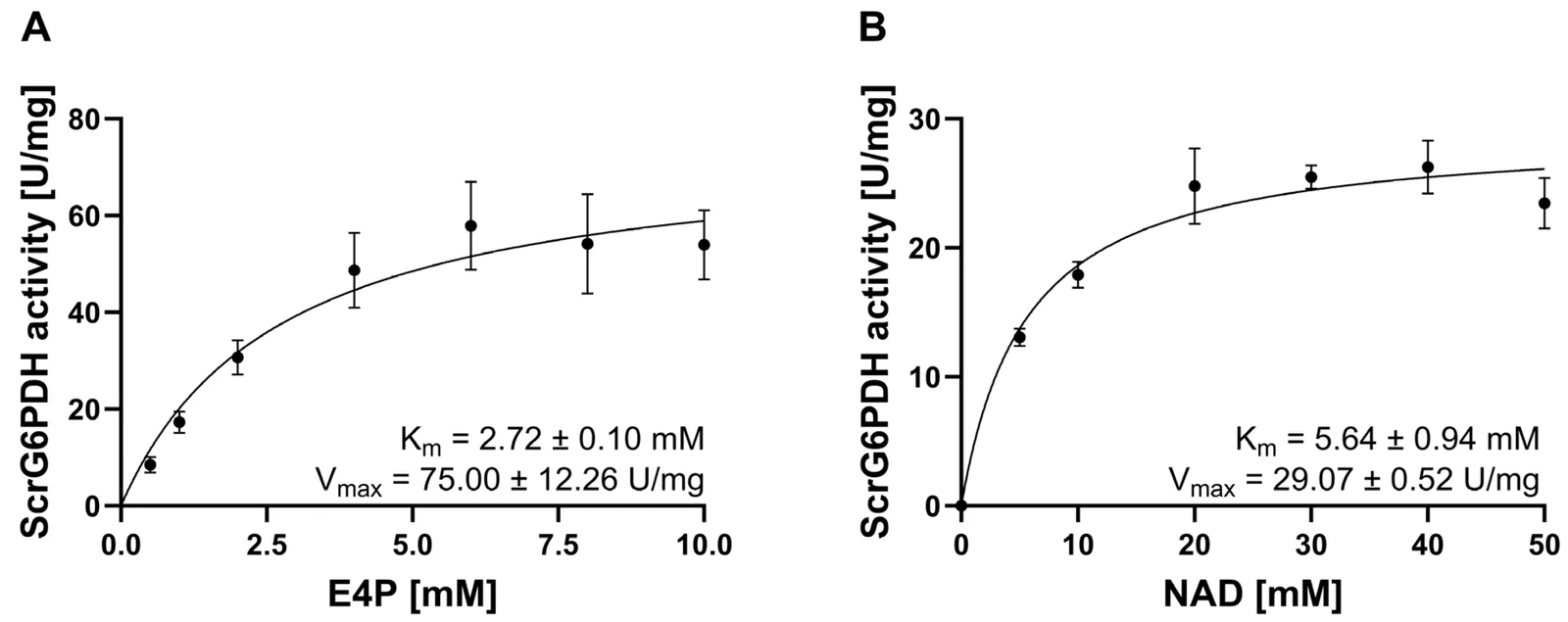
Michaelis–Menten plot. Kinetic parameters, Km and Vmax, of purified G6PDH from *S. cristatus* were determined (**A**) for different E4P concentrations with the NADP concentration fixed at 10 mM, and (**B**) for different NAD concentrations with the G6P concentration fixed at 6 mM. Activities are shown in U/mg protein. Mean values and standard deviations, n = 3.

ScrGapN activity was unaffected by G6P or 6PG, consistent with docking results indicating steric exclusion from the active site and off-site binding (Table 3). G6P and 6PG docked off-site at the vestibule of ScrGapN, where competition with G3P binding is unlikely despite comparable docking scores, consistent with the lack of inhibition. Likewise, ScrG6PDH and Scr6PGDH activities were not affected by each other’s substrates (Figure 6B,C, Table 3). However, both enzymes were significantly inhibited by G3P. Docking supported this, showing favorable binding of G3P in both active sites with docking scores even more favorable than the respective native substrate. However, while the average docking score of 6PG:ScrG6PDH was significantly less favorable than that of the native pair, G6P:ScrG6PDH, the highest-ranked docking pose of G6P:Scr6PGDH had a comparable score (−8.09 kcal/mol) to the native substrate (best score: −8.35 kcal/mol) (Supplementary Table S2). Although this suggested competitive inhibition, no inhibitory effect was observed experimentally. G6P was therefore tested as an alternative substrate and was found to be converted by Scr6PGDH (Supplementary Table S3). In contrast to ScrGapN, both 6PGDH and ScrG6PDH were not impaired by E4P, despite its role in the PPP. Docking predicted comparable, although slightly less favorable, scores for E4P compared to the native substrates. Experimental validation showed that E4P is indeed converted by ScrG6PDH (Figure 7A) but not by Scr6PGDH (Supplementary Table S3), suggesting that docking correctly predicted substrate binding but yielded a false-positive prediction for Scr6PGDH. Kinetic characterization confirmed catalytic activity of ScrG6PDH with E4P, indicating that the predicted binding affinity reflects substrate interaction rather than competitive inhibition. The enzyme exhibited a high turnover rate with E4P (Kcat of 37,084 ± 6062 s^−1^) but a reduced binding affinity (Km = 2.72 ± 0.32 mM) compared to the native substrate G6P. This resulted in a lower overall catalytic efficiency of (21.36 ± 0.23) × 10^7^ M^−1^ s^−1^, which further underlines the structural preference for G6P.

### 3.5. Substrate and Cofactor Specificity of GapN, G6PDH and 6PGDH

To further evaluate the functional relevance of the docking results, substrate and cofactor specificity was investigated experimentally. Given the comparable docking scores for non-native substrates and proximity of oxidation sites to catalytic residues (e.g., G6P docking to 6PGDH), the ability of ScrGapN, ScrG6PDH, and Scr6PGDH to utilize equimolar concentrations of alternative substrates or cofactors was further assessed (Supplementary Table S3). ScrGapN and ScrG6PDH exhibited enzymatic activity exclusively with their native substrates, G3P or G6P, respectively. For ScrGapN, no activity was detected with NAD^+^, indicating a strict dependency on NADP^+^. ScrG6PDH could utilize NAD^+^ as a cofactor, albeit at only about 15% of the activity observed with NADP^+^ at the same concentration. Michaelis–Menten kinetics revealed a substantially lower affinity for NAD^+^ (Km 5.64 ± 0.94 mM; Figure 7B) and a reduced substrate turnover (Kcat = 14,374 ± 257 s^−1^, Table 4) compared to NADP^+^ (Figure 4, Table 4). This culminated in a nearly 50-fold lower catalytic efficiency of (2.55 ± 0.43) × 10^6^ M^−1^ s^−1^, confirming a clear preference for NADP^+^ as the physiological cofactor. For Scr6PGDH, no enzymatic activity was detected when assays were performed with G3P or with NAD^+^ as cofactor. However, G6P was converted as an alternative substrate, reaching approximately 4.5% of the activity measured with 6PG (Supplementary Table S3). Attempts to investigate differential cofactor sensitivity of ScrGapN, ScrG6PDH, and Scr6PGDH by re-docking NADP^+^ and NAD^+^ into the homology models were unsuccessful, as satisfactory binding poses of the cofactors were not obtained. The comparatively large NADP^+^ molecule proved difficult to re-dock to its binding site, as its high internal flexibility hindered the docking algorithm from simultaneously positioning both the adenine- and ribose-nicotinamide moieties correctly.

### 3.6. Growth-Phase Dependent Gene Expression and Activity of NADP Reducing Enzymes

**Figure 8:**
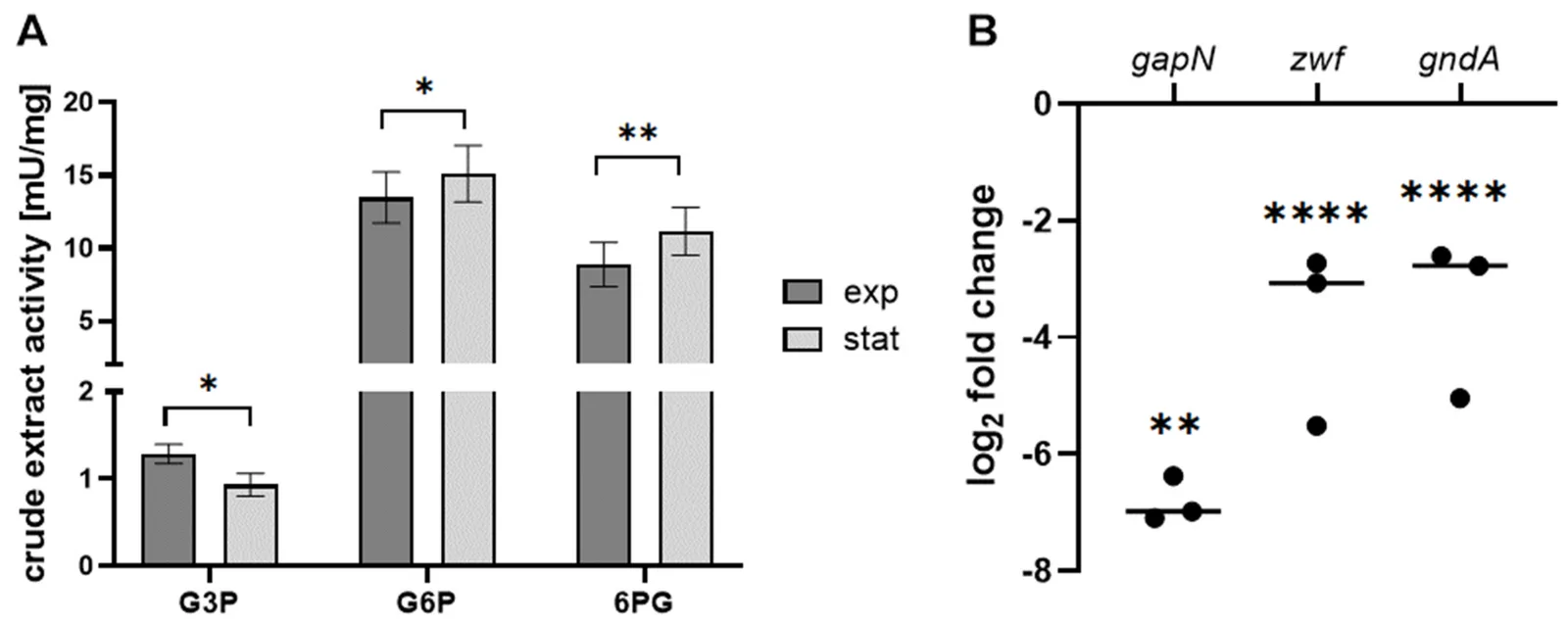
(**A**) Activity of *S. cristatus* protein crude extracts in exponential and stationary growth phases when treated with G3P, G6P, or 6PG, respectively. Activities shown in mU/mg protein crude extract. Mean values and standard deviation, n = 3, paired *t*-test, * *p* < 0.05, ** *p* < 0.01. (**B**) Relative expression of *S. cristatus gapN*, *zwf*, and *gndA* determined using the ΔΔCt method. Gene expression in the stationary phase was compared to that of the exponential phase; both were normalized to 16S rRNA of their respective growth phases. Data are shown as log_2_ fold change. Individual values and median, n = 3, two-way ANOVA, ** *p* < 0.01, **** *p* < 0.0001.

Finally, the physiological relevance of the enzymes was assessed by analyzing their expression and activity during different growth phases. NADP^+^-reducing activity in crude extracts was measured from *S. cristatus* obtained in exponential and stationary growth phases. Overall, crude extracts exhibited lower NADP^+^ reduction activity with G3P than with G6P or 6PG (Figure 8A). NADP^+^ reduction with G6P was considered to reflect combined G6PDH and downstream oxPPP activity, including 6PG conversion by 6PGDH. Activities with G6P and 6PG were slightly yet significantly increased in the stationary phase, whereas transcript levels of *zwf* and *gndA* showed a 3-fold downregulation (Figure 8B). In contrast, G3P-related NADP^+^ reduction and *gapN* transcript levels were both significantly decreased in the stationary phase, with *gapN* showing an approx. 7-fold reduction.

## 4. Discussion

The kinetic characterization of the *S. cristatus* enzymes shows that all three NADP-dependent oxidoreductases display maximal activity under neutral to mildly alkaline conditions, consistent with the pH optima reported for this enzyme class [35,49,50,51].

Despite its substantially higher amino acid sequence identity to *S. pneumoniae* GapN (87%) compared to that of *S. pyogenes* (76%), ScrGapN displayed kinetic parameters that more closely resemble those of *S. pyogenes* (Km (G3P) = 0.58 mM; Km (NADP) = 0.36 mM) than those of *S. pneumoniae* (Km (G3P) = 0.143 mM; Km (NADP) = 0.119 mM) [17,20]. This discrepancy suggests that enzyme kinetics might be shaped not only by sequence conservation but also by metabolic context. Phylogenetically, *S. cristatus* and *S. pneumoniae* are both members of the Mitis group of streptococci, whereas *S. pyogenes* belongs to the pyogenic streptococci [52,53]. As an oral commensal, *S. cristatus* is associated with multispecies dental biofilms, while *S. pneumoniae* primarily colonizes the human nasopharynx, and *S. pyogenes* resides in the human pharynx and skin, indicating that these species each occupy a distinct ecological niche and may therefore face different metabolic demands [23,27,52,54,55,56]. Therefore, the relative contribution of GapN to cellular NADPH generation may differ among these species. Both *S. cristatus* and *S. pneumoniae* encode GapN as well as the oxPPP, while *S. pyogenes* lacks the oxPPP [20]. ScrG6PDH and Scr6PGDH exhibit lower substrate and cofactor affinities than homologous enzymes from *E. coli*, *Pseudomonas* (*P.*) *aeruginosa*, *Klebsiella pneumoniae*, and *Enterococcus faecalis* [49,50,57,58,59], organisms that strongly rely on the oxPPP for NADPH production. Docking scores closely reflected the experimental Km values, with ScrGapN showing the most favorable substrate interactions, followed by Scr6PGDH and ScrG6PDH. These results support both the structural models and the proposed relationship between active-site architecture and substrate affinity. Taken together, the comparatively high Km values and the resulting moderate catalytic efficiencies for all three *S. cristatus* enzymes are consistent with functional redundancy between GapN and the oxPPP, probably reducing selective pressure for maximal catalytic efficiency while increasing metabolic flexibility.

Genome annotation-based comparative analysis of representative streptococci further supports this hypothesis. *S. cristatus* and *S. pneumoniae* encode both GapN and the oxPPP, whereas *S. pyogenes* and *S. dysgalactiae* rely primarily on GapN, and *S. suis* and *S. mitis* encode only the oxPPP (Supplementary Table S4) [60,61,62,63,64,65]. Although *S. pyogenes* and *S. dysgalactiae* additionally encode the NADP-dependent succinate semialdehyde dehydrogenase GabD, its physiological contribution to NADPH production remains unclear because no functional GABA pathway has been described in these species [60,64,66].

Comparison of predicted anabolic pathways suggests that species harboring both GapN and the oxPPP generally encode a broader repertoire of NADPH-dependent biosynthetic enzymes than species relying on a single NADPH-generating pathway (Supplementary Table S4). Core NADPH-dependent enzymes involved in fatty acid biosynthesis, folate metabolism, and thioredoxin-dependent redox homeostasis are conserved throughout the above-mentioned streptococci, whereas the number of predicted NADPH-dependent enzymes involved in amino acid biosynthesis varies considerably. Notably, *S. cristatus* and *S. pneumoniae* encode substantially more of these enzymes than *S. pyogenes*. This observation is consistent with a higher overall NADPH demand in species carrying both NADPH-producing pathways. However, these conclusions should be interpreted with caution as they are based on genome annotations, and the physiological cofactor preferences and metabolic relevance of many enzymes have not been experimentally verified. For example, despite encoding a shikimate dehydrogenase, *S. pyogenes* is auxotrophic for aromatic amino acids [67], illustrating that gene presence alone does not necessarily reflect metabolic activity.

Together, these comparative data suggest that the occurrence of GapN and the oxPPP in *S. cristatus* points toward a metabolic flexibility where NADPH production can be maintained under varying conditions, rather than maximizing the efficiency of a single NADPH-generating pathway.

ScrGapN was highly specific for G3P and strictly dependent on NADP^+^, in agreement with previous studies on GapN from *S. pyogenes* and *S. bovis*, while SpnGapN was shown to accept NAD^+^, though with severely reduced efficiency [17,20,68]. ScrG6PDH also showed strong substrate and cofactor preferences but retained limited activity with E4P and NAD^+^, a promiscuity that has been reported for G6PDH from several bacteria [49,50,58,69,70,71]. In particular, the substitution of NADP^+^ with NAD^+^ led to a substantial decrease in cofactor affinity alongside a significantly reduced turnover number, resulting in a nearly 50-fold drop in catalytic efficiency. Contrary to this, Scr6PGDH showed strict NADP^+^ specificity, consistent with biochemical studies with homologous enzymes from *Leishmania donovani* and *Thermotoga maritima* that strongly prefer NADP^+^ over NAD^+^ [51,72].

Screening for metabolic regulators showed that the three enzymes are largely insensitive to PPP intermediates and salts, consistent with previous studies [16,20]. Structural analyses explain these observations: The narrow ScrGapN active site excludes bulky substrates such as G6P or 6PG. In contrast, the small G3P molecule effectively inhibits ScrG6PDH and Scr6PGDH, consistent with docking predictions. The more accessible active sites of ScrG6PDH and Scr6PGDH permit limited substrate promiscuity, including the utilization of G6P (Scr6PGDH) and E4P (ScrG6PDH). Notably, these experiments were initiated in response to unanticipated docking results, illustrating the value of combining experimental and computational approaches. ATP inhibition of ScrG6PDH agrees with regulatory mechanisms described for G6PDH from *P. aeruginosa*, *Methylomonas* M15, and *Corynebacterium* (*C.*) *glutamicum* [50,73,74,75]. For Scr6PGDH, NADPH inhibition was also observed for 6PGDH from *C. glutamicum* and *E. coli* [75,76]. In contrast, 6PGDH inhibition caused by NADH was not observed in these organisms. The lower inhibitory effect of E4P on ScrGapN, with approx. 60% residual activity observed in our study, compared to only 10.8% for SpyGapN [20], suggests species-specific modulation, potentially arising from differences in global properties of the enzyme rather than active-site mutations, highlighting the subtle electrostatic contributions to inhibitor binding. For ScrG6PDH, E4P served as an alternative substrate where high turnover rates partially compensated for a severely impaired binding affinity, ultimately yielding a lower overall catalytic efficiency than with G6P, consistent with observations for *E. coli* G6PDH [77]. Although these experiments provide insights into enzyme function, intracellular metabolite concentrations, cofactor ratios, and the metabolic state of the cell are likely to influence enzyme activity in vivo. Therefore, the in vitro data do not necessarily reflect the enzyme behavior under physiological conditions.

Overall, docking predictions agreed well with the biochemical data. Most predicted ligand–protein interactions were supported experimentally, and docking scores broadly paralleled the measured Km values. The main exception was E4P binding to Scr6PGDH, illustrating the expected limitations of molecular docking. Similarly, unsuccessful re-docking of NADP^+^ and NAD^+^ likely reflects the difficulty of accurately modeling the binding of large, flexible cofactors using homology models.

Growth-phase-dependent gene expression and enzyme activities further indicate that *S. cristatus* adjusts NADPH production in response to metabolic demands. Reduced GapN activity and transcription in the stationary phase are consistent with decreased anabolic requirements, with similar observations being reported for *S. pyogenes* [20]. The slight increase in G6P- and 6PG-dependent activities observed during the stationary phase suggests that the oxPPP may serve to sustain basal NADPH production in this phase [78].

## 5. Conclusions

In conclusion, the combined structural, biochemical, computational, and comparative analyses suggest that NADPH metabolism in *S. cristatus* is likely optimized for metabolic flexibility rather than maximal catalytic efficiency. The coexistence of GapN and the oxPPP appears to provide a robust framework to maintain general NADPH availability.

## Figures and Tables

**Table 1:** Oligonucleotide primers. Restriction sites are underlined.

Primer	Sequence (5′-3′)	Restriction Sites	PCR Product (bp)
zwf_F	CCCGGATCCTCTTCAAAAGTTATTAT	BamHI	*zwf* gene (1458)
zwf_R	CCCGTCGACTTCTTTATCAGGTTG	SalI	
gndA_F	CCCGGATCCACAAAAGCTAATTTTG	BamHI	*gndA* gene (1419)
gndA_R	CCCGTCGACTTTTTCGTCATACCA	SalI	

**Table 2:** Oligonucleotide primers for qPCR.

Primer	Sequence (5′-3′)
q_16S-rRNA_F	ACCAGAAAGGGACGGCTAAC
q_16S-rRNA_R	TCTACGCATTTCACCGCTAC
q_gapN_F	TGCCCTTATCGGCGGAAATG
q_gapN_R	TGAAAACACCAGCAGGCAGAC
q_zwf_F	TCTACTACTGAAACCTTTGCTTCTGGGG
q_zwf_R	AGTTCTCCAACTTCCTTGCCATTGA
q_gndA_F	CAGAAGACGTAATCGCTTGCCATCC
q_gndA_R	ACCACCAGAAACCCCTGTACCG

**Table 3:** Calculated Kcat values and catalytic efficiency Kcat/Km for *S. cristatus* GapN, G6PDH, and 6PGDH determined for their respective substrates and cofactors. For Kcat/Km, the standard error was calculated via Gaussian error propagation based on the relative errors of Kcat and Km. Mean values ± standard error, n = 3.

Enzyme	Substrate	Cofactor	Kcat [s^−1^]	Kcat/Km [M^−1^ s^−1^]
ScrGapN	G3P		6650 ± 181	(1.71 ± 0.14) × 10^7^
		NADP	6030 ± 315	(2.01 ± 0.35) × 10^7^
ScrG6PDH	G6P		80,630 ± 9231	(5.80 ± 0.71) × 10^7^
	E4P		37,084 ± 6062	(1.36 ± 0.23) × 10^7^
		NADP	84,091 ± 2176	(1.33 ± 0.13) × 10^8^
		NAD	14,374 ± 257	(2.55 ± 0.43) × 10^6^
Scr6PGDH	6PG		12,723 ± 2630	(1.82 ± 0.56) × 10^7^
		NADP	25,273 ± 7006	(9.36 ± 4.33) × 10^7^

**Table 4:** Average docking scores (over three poses per compound) of all active-site binding compounds to *S. cristatus* GapN, G6PDH and 6PGDH with standard deviations. The native enzyme-substrate pairs are highlighted in boldface.

Compound	Average Docking Score: ScrGapN [kcal/mol]	Average Docking Score: ScrG6PDH [kcal/mol]	Average Docking Score: Scr6PGDH [kcal/mol]
G3P	**−8.00** ± **0.03**	−6.32 ± 0.07	−7.97 ± 0.14
G6P	−7.21 ± 0.03 *	**−6.21** ± **0.03**	−6.98 ± 1.27
6PG	−7.10 ± 0.05 *	−5.43 ± 0.08	**−7.75** ± **0.53**
E4P	−6.93 ± 0.16	−6.05 ± 0.11	−7.59 ± 0.19
S7P	−5.84 ± 0.05 *	−3.70 ± 0.16	−5.56 ± 0.11
Pyruvate	−5.08 ± 0.35	−3.58 ± 0.21	−5.57 ± 0.11

* Off-site binding at a location other than the active site.

## Data Availability

The raw data supporting the findings of this study have been deposited in the Zenodo repository and include experimental datasets (biological replicate data underlying figures and tables) and computational modeling data. The data can be accessed via https://doi.org/10.5281/zenodo.21156586.

## Supplementary Materials

The following supporting information can be downloaded at: https://www.mdpi.com/article/10.3390/biom16081212/s1. Figure S1: Purification of *S. cristatus* GapN, G6PDH and 6PGDH. SDS-PAGE gels were loaded with PageRuler Prestained Protein Ladder (M), pre- (1) and post-induction samples (2), flow through fraction (3), wash fractions (4 and 5), and elution fractions (6) of the StrepTactin affinity chromatography. To detect proteins carrying a Strep-tag, Strep-tag alkaline phosphatase conjugate and NBT/BCIP were used. The left panel shows Coomassie-stained SDS-PAGE gels, the right panel shows corresponding Western blot membranes; Table S1. Quality descriptors of the homology models of the *S. cristatus* GapN, G6PDH and 6PGDH enzymes. In addition to the sequence identity between the template and the target (SeqID), the Global QMeanDisCo score as provided by SWISS-Model (ranging from 0: bad to 1: excellent), and the number of amino acid residues counting as Ramachandran outliers in the modeled structure are provided; Table S2. Highest-ranked docking of all active-site binding compounds to *S. cristatus* GapN, G6PDH and 6PGDH. The native enzyme-substrate pairs are highlighted in bold face; Table S3. Specific enzyme activities [%] measured in *S. cristatus* GapN, G6PDH, 6PGDH in presence of NAD or G3P, G6P and 6PG as substrates compared to standard assay conditions (100%). Mean values ± standard deviation, n = 3; Table S4. Overview of the presence (+) or absence (−) of NADP^+^-reducing (shaded in gray) or NADPH-oxidizing enzymes from selected metabolic pathways in *S. cristatus* (Scr), *S. pneumoniae* (Spn), *S. pyogenes* (Spy), *S. dysgalactiae* ssp. *equisimilis* (Seq), *S. suis* (Ssu) and *S. mitis* (Smi) based on KEGG annotations.
