# Supplementary material for: Catalytic Properties of NADP-Reducing Enzymes from Streptococcus cristatus ATCC 51100
Source: Biomolecules. 2026 Aug 20;16(8):1212. doi: 10.3390/biom16081212 (PMC13511739; doi:10.3390/biom16081212)
Supplement: Supplementary file 1 [file biomolecules-16-01212-s001.zip › biomolecules-4446615-supplementary.pdf]

Supplementals

Figures

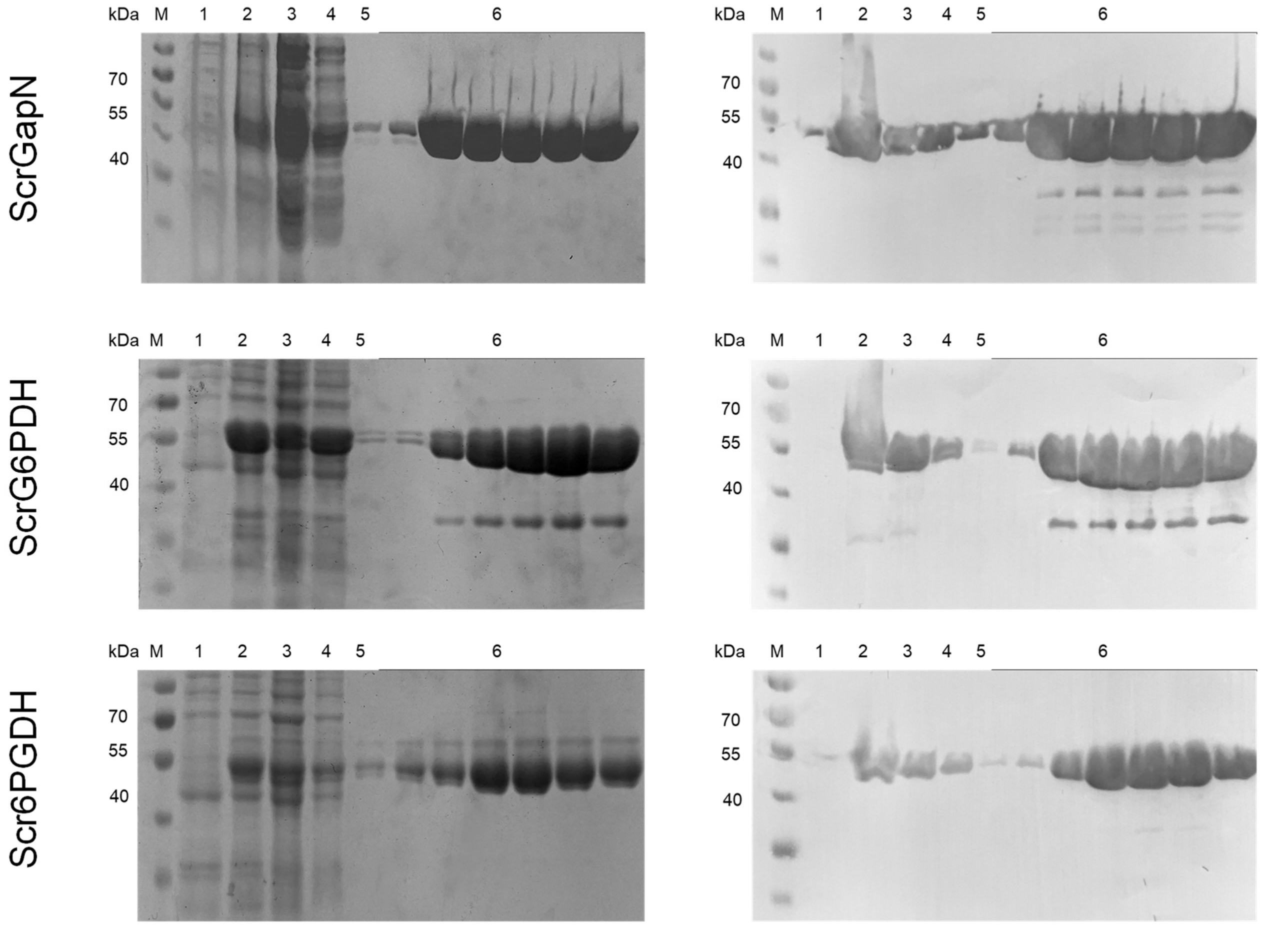

Supplementary Figure S1. Purification of *S. cristatus* GapN, G6PDH and 6PGDH. SDS-PAGE gels were loaded with PageRuler Prestained Protein Ladder (M), pre- (1) and post-induction samples (2), flow through fraction (3), wash fractions (4 and 5), and elution fractions (6) of the StrepTactin affinity chromatography. To detect proteins carrying a Strep-tag, Strep-tag alkaline phosphatase conjugate and NBT/BCIP were used. The left panel shows Coomassie-stained SDS-PAGE gels, the right panel shows corresponding Western blot membranes.

Tables

Supplementary Table S1. Quality descriptors of the homology models of the *S. cristatus* GapN, G6PDH and 6PGDH enzymes. In addition to the sequence identity between the template and the target (SeqID), the Global QMeanDisCo score as provided by SWISS-Model (ranging from 0: bad to 1: excellent), and the number of amino acid residues counting as Ramachandran outliers in the modeled structure are provided.

**Model**	**ScrGapN**	**ScrG6PDH**	**Scr6PGDH**
**Template Structure**	2QE0	1H9A	2IYO
**Global QMEANDisCo**	0.89 $\pm$ 0.05	0.78 $\pm$ 0.05	0.83 $\pm$ 0.05
**SeqID [%]**	75.90	45.21	74.10
**Ramachandran outliers [# residues]**	0	1	0

Supplementary Table S2**.** Highest-ranked docking of all active-site binding compounds to *S. cristatus* GapN, G6PDH and 6PGDH. The native enzyme-substrate pairs are highlighted in bold face.

**Compound**	**Highest-ranked docking score: ScrGapN [kcal/mol]**	**Highest-ranked docking score: ScrG6PDH [kcal/mol]**	**Highest-ranked docking score: Scr6PGDH [kcal/mol]**
**G3P**	**-8.28**	-6.35	-7.86
**G6P**	-7.24*	**-6.23**	-8.09
**6PG**	-7.15*	-5.52	**-8.35**
**E4P**	-7.11	-6.14	-7.78
**S7P**	-5.90*	-3.87	-5.63
**Pyruvate**	-5.43	-3.76	-5.68

**Off-site* binding at a location other than the active site

Supplementary Table S3. Specific enzyme activities [%] measured in *S. cristatus* GapN, G6PDH, 6PGDH in presence of NAD or G3P, G6P and 6PG as substrates compared to standard assay conditions (100 %). Mean values ± standard deviation, n = 3.

**Enzyme**	**substrates**				**cofactors**	
	**2 mM G3P**	**6 mM G6P**	**6 mM 6PG**	**2 mM E4P**	**10 mM NADP**	**10 mM NAD**
**ScrGapN**	100	0	0	0	100	0
**ScrG6PDH**	0	100	0	27.0 ± 1.9	100	14.8 ± 1.1
**Scr6PGDH**	0	4.5 ± 0.1	100	0 *	100	0

* for 6PGDH, up to 10 mM E4P were tested, however no enzymatic activity could be detected.

Supplementary Table S4. Overview of the presence (+) or absence (-) of $NADP^+$-reducing (shaded in gray) or NADPH-oxidizing enzymes from selected metabolic pathways in *S. cristatus* (Scr), *S. pneumoniae* (Spn), *S. pyogenes* (Spy), *S. dysgalactiae* ssp. *equisimilis* (Seq), *S. suis* (Ssu) and *S. mitis* (Smi) based on KEGG annotations.

**Pathway**	**Enzyme**	**EC number**	**Scr**	**Spn**	**Spy**	**Seq**	**Ssu**	**Smi**
oxPPP	Glucose-6-phosphate dehydrogenase	1.1.1.49	+	+	-	-	+	+
	Phosphogluconate dehydrogenase	1.1.1.44	+	+	-	-	+	+
Glycolysis	Non-phosphorylating glyceraldehyde-3-phosphate dehydrogenase	1.2.1.9	+	+	+	+	-	-
GABA metabolism	Succinic semialdehyde dehydrogenase	1.2.1.79	-	-	+	+	-	-
Fatty Acid Biosynthesis	3-oxoacyl-[acyl-carrier-protein] reductase	1.1.1.100	+	+	+	+	+	+
	Enoyl-[acyl-carrier-protein] reductase	1.3.1.10	-	-	-	-	-	-

Aromatic Amino Acid Biosynthesis	Shikimate dehydrogenase	1.1.1.25	+	+	+	-	-	-
	Prephenate dehydrogenase	1.3.1.13	-	-	-	-	-	-
	Arogenate dehydrogenase	1.3.1.78	-	+	-	-	+	+
Biosynthesis of other Amino Acids	Aspartate semialdehyde dehydrogenase	1.2.1.11	+	+	-	+	+	+
	4-hydroxy-Tetrahydrodipicolinat reductase	1.17.1.8	+	+	-	-	+	+
	Homoserine dehydrogenase	1.1.1.3	+	+	-	+	+	+
	ɣ-Glutamylphosphate reductase	1.2.1.41	-	-	+	+	+	+
	Pyrrolin-5-carboxylate reductase	1.5.1.2	+	-	+	+	+	+
	Glutamate dehydrogenase	1.4.1.4	+	+	-	+	+	+
Others	Thioredoxin reductase	1.8.1.9	+	+	+	+	+	+
	Dihydrofolate reductase	1.5.1.3	+	+	+	+	+	+
